# Development of a complete human anti-human transferrin receptor C antibody as a novel marker of oral dysplasia and oral cancer

**DOI:** 10.1002/cam4.267

**Published:** 2014-06-02

**Authors:** Kentaro Nagai, Shingo Nakahata, Shunsuke Shimosaki, Tomohiro Tamura, Yuudai Kondo, Takashi Baba, Tomohiko Taki, Masafumi Taniwaki, Gene Kurosawa, Yukio Sudo, Seiji Okada, Sumio Sakoda, Kazuhiro Morishita

**Affiliations:** 1 Division of Oral and Maxillofacial Surgery, Medicine of Sensory and Motor Organs, University of MiyazakiMiyazaki, Japan; 2 Division of Tumor and Cellular Biochemistry, Department of Medical Sciences, Faculty of Medicine, University of MiyazakiMiyazaki, Japan; 3 Department of Hematology and Oncology, Kyoto Prefectural University of MedicineKyoto, Japan; 4 Perseus Proteomics Inc.Tokyo, Japan; 5 Division of Hematopoiesis, Center for AIDS Research, Kumamoto UniversityKumamoto, Japan

**Keywords:** Biomarker, iron metabolism, OSCC, therapeutic antibody, transferrin receptor C

## Abstract

Oral squamous cell carcinoma (OSCC) is the sixth most common cancer worldwide. Up to 20% of oral dysplasia cases have been suggested to undergo malignant transformation to OSCC; however, there are no methods to predict OSCC development. In this study, to identify the genes associated with oral dysplasia progression, we performed genomic copy number analyses of genomic DNA samples isolated from primary oral dysplasia and OSCC via the microdissection method and found elevated expression of transferrin receptor C (TfR1/TFRC) with genomic amplification in oral dysplasia and OSCC. The expression rate of TFRC in OSCC was significantly higher than that in dysplasia, suggesting that OSCC disease progression might be related to TFRC expression. Additionally, we investigated the in vitro and in vivo impacts of a newly established anti-human TFRC monoclonal antibody, which was isolated from a human cDNA library using the phage-display method, on cell proliferation and survival. The anti-TFRC antibody blocked the interaction between transferrin and TFRC and consequently inhibited iron uptake, leading to the iron deprivation-mediated suppression of cell growth and induction of apoptosis. Moreover, we demonstrated that the anti-TFRC antibody efficiently inhibited tumor growth in a murine xenograft OSCC model. Therefore, we suggest our developed complete human anti-human TFRC antibody as a useful, novel treatment for oral dysplasia and OSCC.

## Introduction

Oral squamous cell carcinoma (OSCC) is the sixth most prevalent tumor type worldwide 1,2. The prognosis of OSCC patients is poor, and oral dysplasia is a relatively common precursor of oral cancer 3,4; however, the early diagnosis and effective treatment of oral dysplasia and oral cancer have not been established 5. Oral carcinogenesis is a multifactorial process, and chronic exposure to carcinogens, such as tobacco, is a significant factor in OSCC development. In previous studies, we and others have performed genome-wide gene copy number analyses of OSCC surgical specimens, in which gains and losses of many chromosomal segments were frequently detected 6,7. Furthermore, many oncogenes and suppressor genes, including AIM2 and IFI16, have been identified 7. On the other hand, a few oncogenic-responsive genes were identified in oral dysplasia, including p53 and ALCAM 8. Therefore, to identify the responsive genes for oral dysplasia progression, in this study, we performed a comparative genomic hybridization (CGH) analysis of DNA from oral dysplasia and OSCC samples collected by microdissection and found higher expression levels of transferrin receptor C (TfR1/TFRC) with genomic amplification in oral dysplasia and OSCC.

TFRC plays a crucial role in the cellular uptake of iron, and cellular iron deficiency arrests cell growth and leads to cell death 9. In malignant tissues, TFRC is expressed more abundantly than in their normal tissue counterparts 10 because cancer cells require large amounts of iron to maintain their high cell proliferation rates. Therefore, TFRC is an attractive target for immunotherapy and the delivery of cytotoxic agents due to its increased expression on malignant cells compared to normal cells 11. We previously established a procedure for the comprehensive identification of tumor-specific antibodies via the extensive isolation of human monoclonal antibodies (mAbs) using the phage-display method 12. Several antibody libraries were screened, and a large number of human mAbs that bound to the surfaces of tumor cells were isolated. In the final stage of the screening procedure, 488 different monoclonal clones and 29 different tumor-associated antigens were obtained, including antibodies against human TFRC 13.

In this study, we investigated the expression levels of TFRC in oral dysplasia and OSCC cells and the in vitro and in vivo impacts of a newly established human anti-TFRC monoclonal antibody on cell proliferation and survival. Herein, we show that TFRC is highly expressed in both oral dysplasia and OSCC. The anti-TFRC antibody blocked the interaction between transferrin and TFRC and, consequently, iron uptake, leading to iron deprivation and the inhibition of cell growth and the induction of apoptosis. More importantly, we demonstrated that the anti-TFRC antibody efficiently inhibited OSCC tumor growth in a murine xenograft model. Thus, we suggest this newly established anti-TFRC antibody as a useful and novel treatment for oral dysplasia and OSCC.

## Materials and Methods

### Cell lines

Eight human OSCC cell lines (Ca922, HO-1-u-1, HSC2, HSC3, HSC4, SAS, HSQ89, and Sa-3) were purchased from the RIKEN BioResource Center (Ibaraki, Japan). The human keratinocyte HaCaT cell line was obtained from the Cell Line Service (Eppelheim, Germany) as a control. All cell lines were maintained in appropriate media (RPMI-1640 or Dulbecco's modified Eagle's medium) supplemented with 10% fetal bovine serum, 100 U/mL penicillin, and 100 *μ*g/mL streptomycin.

### Laser microdissection

For RNA extraction, 6–8 *μ*m frozen sections were placed at room temperature for 10–20 min and fixed in 70% ethanol for 15 sec. After washing two times, the sections were stained with 0.05% Toluidine Blue (Wako, Japan). Finally, the sections were washed with water and then air dried. The sections were microdissected with a Leica CTR MIC (Leica Microsystems, Oberkochen, Germany). The target areas were normal epithelium, epithelial dysplasia, and OSCC from the cancer patients. Prior to microdissection, we removed the keratinized area from the well-differentiated regions that form the tumor mass. The number of microdissected cells per sample was estimated to be ∼500–2000. For DNA extraction, the sections were fixed in 70% ethanol, and the number of microdissected cells was ∼10,000–30,000.

### High-density SNP array-CGH analysis

Genomic DNA was isolated from eight OSCC tissue samples and eight oral dysplasia tissue samples and analyzed using GeneChip SNP genotyping microarrays (Genome-Wide Human SNP Nsp Array; Affymetrix, Santa Clara, CA). After the appropriate normalization of the mean array intensities, the signal ratios between the tumor samples and anonymous normal references were calculated in an allele-specific manner. Allele-specific copy numbers were inferred from the observed signal ratios based on the hidden Markov model using CNAG/AsCNAR software (Genome Laboratory, University of Tokyo, Tokyo, Japan).

### Antibodies

Several anti-human TFRC antibodies were isolated using phage-display technology as previously described 12–14. After screening these antibodies, the Fc portion was used to reconstruct a complete human IgG form of the monoclonal antibody. The complete IgG form was used for the entire OSCC treatment experiment. The commercial antibody against TFRC and caspase-3 for immunoblot was obtained from Cell Signaling Technologies (Beverly, MA; catalog no. 9251).

### Reverse-transcription polymerase chain reaction

The levels of TFRC, ferritin light polypeptide (FTL), ferritin heavy polypeptide 1 (FTH1), and *β*-actin mRNA in the OSCC cell lines were measured by RT-PCR. Briefly, total RNA was extracted using Trizol (Invitrogen, Carlsbad, CA), and 1 mg of total RNA was reverse transcribed to obtain first-strand cDNA using an RNA-PCR kit (Takara-Bio Inc., Tokyo, Japan). cDNA fragments were amplified by PCR using specific primers. The primers used are as follows: TFRC forward, 5′-CTCACTTTAGACAATGCTGC-3′, reverse, 5′-CTCATGACACGATCATTGAG-3′; FTL forward, 5′-GCGTCTCCTGAAGATGCAAA-3′, reverse, 5′-AGGAAGTGAGTCTCCAGGAAGT-3′; FTH1 forward, 5′-CCCCCATTTGTGTGACTTCAT-3′, reverse, 5′-GCCCGAGGCTTAGCTTTCATT-3′; and *β*-actin forward, 5′-GACAGGATGCAGAAGGAGATTACT-3′, reverse, 5′-TGATCCACATCTGCTGGAAGGT-3′.

### Quantitative real-time PCR analysis

Quantitative real-time RT-PCR was performed with SYBR Green PCR Master Mix (Applied Biosystems, Foster City, CA) using an ABI PRISM 7000 Sequence Detection System (Applied Biosystems). The amplification data were analyzed with ABI PRISM 7000 SDS software (Applied Biosystems), converted into cycle numbers at a set cycle threshold (Ct values) and quantified relative to a standard. HSQ-89 RNA was used as a standard in all experiments. To normalize the amounts of input cDNA, the relative amount of the generated product was divided by the relative amount of *β*-actin. All samples were analyzed in duplicate. The primers used are as follows: TFRC forward, 5′-CATCAGCCTCCTGGTTATGG-3′, reverse, 5′-AAATGCCTCCGCTTATGTTG-3′; and *β*-actin forward, 5′-GACAGGATGCAGAAGGAGATTACT-3′ and reverse, 5′-TGATCCACATCTGCTGGAAGGT-3′.

### Flow cytometry analysis

The cells were incubated with the anti-TFRC antibody on ice for 1 h. They were then washed three times and incubated with a phycoerythrin-labeled streptavidin antibody on ice for 1 h. After washing three times, the treated cells were analyzed on a FACScan flow cytometer (Becton Dickinson, San Jose, CA).

### Generation of OSCC cell lines expressing TFRC

Full-length TFRC cDNA was isolated by RT-PCR using total RNA from human OSCC HSC3 cells as a template and subcloned into the p3XFLAG-myc-CMV-26 expression vector (Sigma-Aldrich, St. Louis, MO) to generate pFLAG-TFRC. HSC4 and HaCaT cells were transiently transfected with this construct using Hillymax reagents (Dojindo Laboratories, Kumamoto, Japan) following the manufacturer's recommendations. Stable cell lines were established by G418 selection.

### Generation of OSCC cell lines expressing shTFRC

A DNA-based small hairpin (sh) RNA expression vector (pSIREN-retroQ-ZsGreen plasmid, Takara-Bio Inc.) was used in the TFRC knockdown experiments. The following sequence was cloned into the BamHI-EcoRI site of the vector to create a shRNA against human TFRC: 5′-GATCGAACAGTCATGTGGAGATGAAATTCAAGAGATTTCATCTCCACATGACTGTTTTTTTTACGCGTG-3′ and 5′-AATTCACGCGTAAAAAAAACAGTCATGTGGAGATGAAATCTCTTGAATTTCATCTCCACATGACTGTTA-3′. pSIREN-retroQ-ZsGreen plasmid (mock) indicates transfection of a shRNA mock expression vector. The OSCC cell line SAS was transiently transfected using the Nucleofector Kit (Amaxa, Gaithersburg, MD) according to the manufacturer's recommendations. After 48 h of transfection, ZsGreen-positive SAS cells were sorted with a JSAN cell sorter (Bay Bioscience, Kobe, Japan).

### Cell proliferation assay

OSCC cells (2 × 10^4^ cells/mL) were incubated with various concentrations of the anti-TFRC antibody in complete medium at 37°C and 5% CO_2_. Cell proliferation was measured by the methyl thiazolyl tetrazolium (MTT) assay using cell counting kit 8 (Dojindo). Each experiment was performed three times, and typical results are shown.

### Apoptosis assay

Apoptosis assays were performed using the Apoptosis Detection kit (MBL, Nagoya, Japan) according to the manufacturer's protocol. HSC4 and SAS cells (2 × 10^5^ cells/mL) were incubated with anti-TFRC (1.0 mg/mL) in RPMI1640 with 10% serum at 37°C and 5% CO_2_ for 72–96 h. The cells were washed and resuspended in binding buffer at a concentration of 1 × 10^6^ cells/mL. The suspension was then incubated with 5 mL Annexin-V and 5 mL propidium iodide for 15 min at room temperature in the dark. The samples were analyzed using a FACScan flow cytometer (Becton Dickinson). The Annexin-V-positive cells were considered to have undergone apoptotic cell death.

### Cell cycle assay

Cells were adjusted to equal numbers of 2 × 10^6^, fixed in ice-cold 70% ethanol overnight and then stored at −20°C. Twenty-four hours prior to flow cytometry analysis, the samples were resuspended in phosphate-buffered saline (PBS) containing 25 *μ*g/mL DNase-free RNase and 10 *μ*L propidium iodide for staining in the dark. DNA content was determined using a FACScan flow cytometer and ModFit LT software (Becton Dickinson).

### Cytotoxicity assays

The complement-dependent cytotoxicity (CDC) and antibody-dependent cellular cytotoxicity (ADCC) activities of anti-TFRC were measured by a lactate dehydrogenase (LDH)-releasing assay using a cytotoxicity detection kit (Roche, Indianapolis, IN). Cells, 1 × 10^4^, of various cell lines (HaCaT, HSC2, HSC3, HSC4, and SAS) were incubated with various concentrations of the anti-TFRC antibody and human serum as the source of complement at a dilution of 1:32 for the CDC assay or with various concentrations of the anti-TFRC antibody and human mononuclear cells from peripheral blood as effector cells (effector:target, 50:1) for the ADCC assay. After an additional incubation for 4 h at 37°C, the amount of LDH released into the medium was determined using a cytotoxicity detection kit. The maximum LDH release was measured by lysing cells with 2% Triton X-100. The percentage of specific lysis was calculated according to the following formula: CDC% specific lysis = 100 × (*E* − *S*)*/*(*M* − *S*), where *E* is the absorbance of the experimental well, *S* is the absorbance in the absence of monoclonal antibody (cells were incubated with medium and complement alone), and *M* is the maximum release from the target cells (activity released from target cells lysed with 2% Triton X-100); ADCC% specific lysis = 100 × (*E* − *S*_E_ − *S*_T_)/(*M* − *S*_E_), where *E* is the experimental release (supernatant activity of target cells incubated with antibody and effector cells), *S*_E_ is the spontaneous release in the presence of effector cells with antibody (supernatant activity of target cells incubated with effector cells), *S*_T_ is the spontaneous release from the target cells (supernatant activity of target cells incubated with medium alone), and *M* is the maximum release from the target cells (activity released from target cells lysed with 2% Triton X-100).

### Transferrin internalization assay

To evaluate the uptake of transferrin into OSCC cells, HSC4 and SAS cells were incubated in serum-free medium at 37°C for 2 h. After the cells were harvested and washed, they were incubated with 50 mg/mL Alexa Fluor 647-conjugated human transferrin (Invitrogen) in binding buffer (RPMI1640 containing 20 mmol/L 4-(2- hydroxyethyl)-1-piperazineethanesulfonic acid (HEPES) pH 7.4, 1% BSA) on ice for 30 min. After washing to remove any unbound transferrin, the OSCC cells were incubated in RPMI1640 with 10% fetal bovine serum at 37°C for the indicated times. After incubating the cells in prechilled acidic buffer (20 mmol/L 2-(N-morpholino) ethanesulfonic acid (MES) pH 5, 130 mmol/L NaCl, 50 mmol/L deferoxamine, 2 mmol/L CaCl_2_, and 0.1% bovine serum albumin (BSA)) on ice for 20 min and then washing three times, the fluorescence intensity of the internalized transferrin in the OSCC cell population was determined using a FACSCalibur flow cytometer (Becton Dickinson).

### Xenograft tumors

Six- to eight-week-old, female, Rag-2/Jak3 double-deficient (Rag-2^−/−^Jak3^−/−^) Balb/c mice 15 were given a single subcutaneous injection of 5 × 10^6^ SAS cells suspended in 100 *μ*L PBS. When the subcutaneous tumors reached an average size of 100–150 mm^3^, the mice were intravenously injected with either PBS or the anti-TFRC antibody (7.5 or 15 mg/kg) two times per week for 3 weeks. The length, width, and height of each tumor were measured with a caliper twice per week and used to calculate the tumor volume.

## Results

### High TFRC expression in OSCC

**Figure 1:**
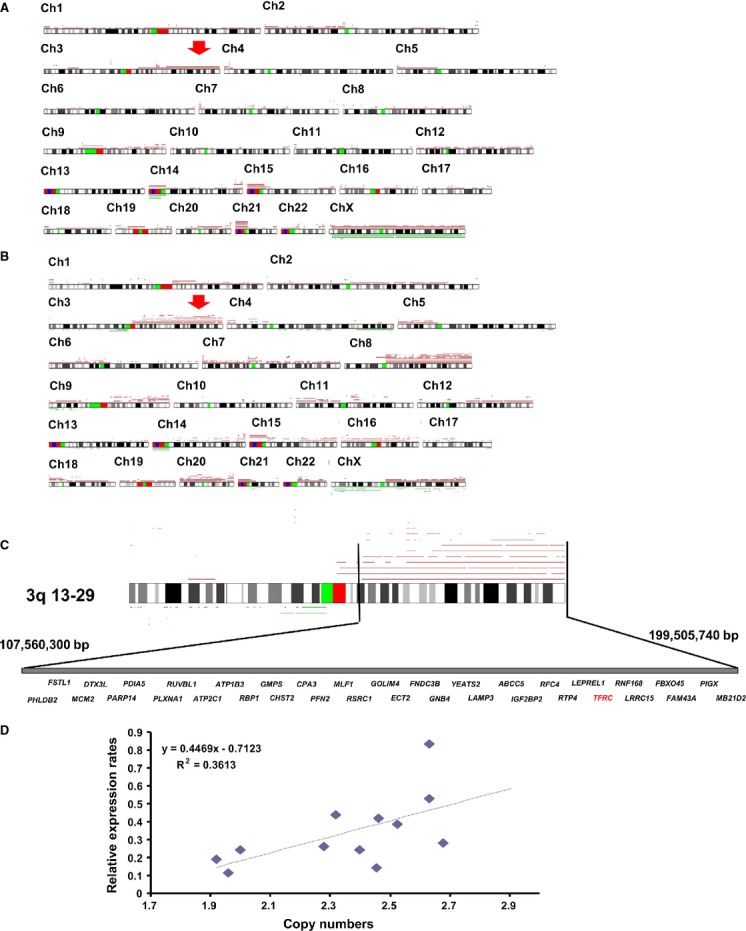
DNA copy number analysis of oral dysplasia and oral squamous cell carcinoma (OSCC) showed an amplified genomic region at chromosome 3q. Recurrent genetic changes are depicted according to the copy number analyzer for the GeneChip (CNAG) output of the single-nucleotide polymorphism array analysis of eight oral dysplasia and eight OSCC samples. A summary of all of the identified genetic alteration patterns in the (A) oral dysplasia and (B) OSCC samples is shown. Regions with copy number gains are indicated by red lines and losses are indicated by green lines. Red arrows show the common amplified regions of chromosome 3q in the dysplasia and OSCC samples. (C) A precise genomic map of the amplified regions of 3q. Several genes in the TFRC region were identified in the OSCC expression profiles as being expressed at levels that were more than twofold higher than those in the control oral tissues. (D) Scatter plot of the DNA copy number versus the TFRC mRNA expression rate. Correlations were quantified with Spearman's rank correlation coefficient. TFRC, transferrin receptor C.

To identify novel therapeutic targets in OSCC, we previously performed a high-density single nucleotide polymorphism (SNP) array analysis of 28 OSCC tumor samples using an Affymetrix Human Mapping 250K Sty Array (GSE34507; Affymetrix) 9. In this study, the genomic copy numbers in eight oral dysplasia samples and eight OSCC tumor samples were analyzed by high-density SNP Array (Affymetrix Human Mapping 250K Nsp Array, GSE51265) after isolating the tumor samples with a Laser Microdissection Capture system (Leica, Wetzlar, Germany). When comparing the copy number differences between the dysplasia and OSCC samples, we found a commonly amplified region at chromosome 3q23-29 and a commonly deleted region at chromosome 14p (Fig.1A–C). To identify candidate tumor-related genes within both regions, we analyzed a data set from the NCBI Gene Expression Omnibus series GSE30874 (http://www.ncbi.nlm.nih.gov/geo/), which contains the gene expression profiles of 167 primary tumors and 17 oral dysplasia samples from OSCC patients and 45 oral tissue samples from healthy volunteers 16. Among the several hundred genes in the amplified regions at chromosome 3q, 35 genes were identified with more than twofold higher expression in OSCC and 1.5-fold higher expression in oral dysplasia (*P* < 0.05; Table S1 and Fig.1C), while no genes were identified in the deleted region at chromosome 14p. Among these genes, three gene products (FNDC3B, RTP4, and TFRC) were suspected to be expressed outside the cellular membrane. We concentrated on the analysis of TFRC (transferrin receptor 1, transferrin receptor C, CD71) as a putative marker receptor for OSCC because we had previously developed several anti-human TFRC antibodies 13. There was a positive correlation (*R* = 0.3613) between the TRFC expression levels and the TFRC copy numbers in oral dysplasia and OSCC (Fig.1D).

**Figure 2:**
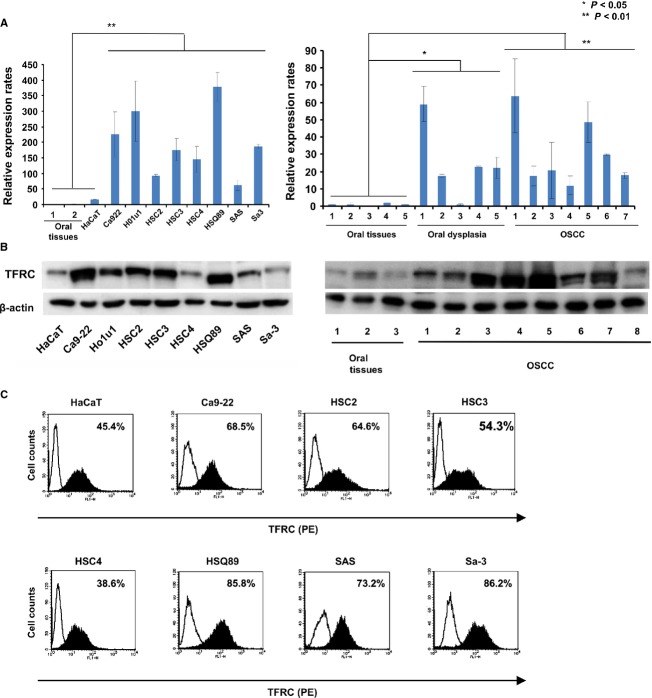
TFRC expression in OSCC. (A) The TFRC mRNA level was examined in five primary oral dysplasia tissues, eight OSCC primary tissues, and eight OSCC cell lines (Ca9-22, Ho1u1, HSC2, HSC3, HSC4, HSQ89, SAS, and Sa-3) by quantitative real-time PCR. mRNA samples from the oral tissues of normal healthy volunteers were used as controls. *P < 0.05, **P < 0.01. (B) TFRC expression was evaluated in primary OSCC tissues compared with normal oral tissues (right panel) and eight OSCC cell lines compared with control keratinocyte HaCaT cells (left panel) by immunoblotting. (C) TFRC expression was evaluated on OSCC cells and control HaCaT cells by flow cytometry using the anti-TFRC antibody. The cells were stained with the phycoerythrin-labeled anti-TFRC antibody, followed by flow cytometry analysis. The figure shows representative flow cytometry histogram profiles of the control keratinocyte cell line (HaCaT) and seven OSCC cell lines (Ca9-22, HSC2, HSC3, HSC4, SAS HSQ89, SAS, and Sa-3). Open histograms represent cells stained with IgG isotype controls, and filled histograms indicate cells stained with the anti-TFRC antibody. *P < 0.01. OSCC, oral squamous cell carcinoma; TFRC, transferrin receptor C.

When we evaluated the expression values for iron-associated genes in the OSCC microarray data set (GSE30784), TFRC and TF (transferrin) showed significant differences between the OSCC and control tissues (Fig. S1A). Therefore, to evaluate TFRC expression in oral dysplasia and OSCC, five cases of oral dysplasia, eight cases of primary OSCC tumors, five oral tissues from healthy volunteers, and eight OSCC cell lines were initially analyzed to determine the expression levels of TFRC and other genes related to iron metabolism (FTL and FTH1) by RT-PCR and quantitative real-time PCR. The TFRC expression level in the OSCC cell lines and OSCC patient tissues was significantly higher than that in the oral tissues from healthy volunteers and the keratinocyte cell line HaCaT (*P* < 0.05; Figs. S1B and C, 2A). In addition, we evaluated the expression level of the TFRC protein in ten primary OSCC tissues and eight OSCC cell lines compared to three oral tissue samples and HaCaT cells by immunoblot analysis using a commercial TFRC antibody (Fig.2B). As shown in Figure2B, TFRC was strongly expressed in the primary OSCC tissues and OSCC cell lines compared with the control tissues and HaCaT cells. Additionally, we evaluated surface expression of TFRC by flow cytometry analysis using a phycoerythrin-labeled anti-human TFRC antibody (Fig.2C). As a control, the HaCaT cell line showed a relatively low percentage of TFRC-positive cells (46.5%) with a relatively low mean fluorescence intensity (MFI; 44.8). However, the OSCC cell lines had higher percentages of TFRC-positive cells (mean, 73.93%) with a higher MFI (mean, 81.96).

**Figure 3:**
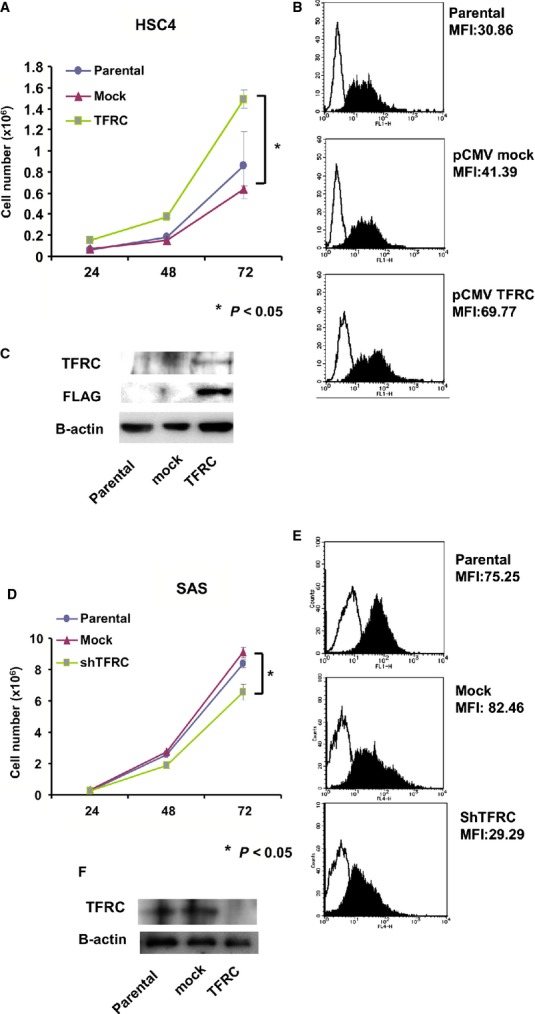
Effects of TFRC knockdown and overexpression on OSCC cell growth. (A) A TFRC expression vector was introduced into HSC4 cells, and cell growth was analyzed by MTT assay. The data are shown as the mean ± SD of triplicate samples. *P < 0.05 versus parental HSC4 cells. The TFRC expression level in parental, mock-, and TFRC-transfected HSC4 cells was determined by FACS (B) analysis and (C) immunoblotting. (D) Retroviral vectors containing TFRC shRNA or mock shRNA (control) were transfected into SAS cells. Forty-eight hours after transfection, the ZsGreen-positive cells were sorted and cell growth was analyzed by MTT assay. The data are shown as the mean ± SD of triplicate samples. *P < 0.05 versus parental SAS cells. OSCC, oral squamous cell carcinoma; TFRC, transferrin receptor C; MTT, methyl thiazolyl tetrazolium.

Next, to evaluate whether the OSCC cell growth rate depends on the level of TFRC expression, we introduced a TFRC expression vector into HSC4/OSCC cells; we used this cell line because its TFRC expression level was relatively lower than that in the other OSCC cell lines. Conversely, an expression vector with TFRC-specific shRNA (shTFRC) was introduced into SAS/OSCC cells, as the level of TFRC expression was relatively higher in this cell line compared to the other OSCC cell lines. After introducing the TFRC expression vector into the HSC4 cells, the growth rate of the HSC4/TFRC cells clearly increased more than that of the mock vector-transfected or parental cells (Fig.3A), and the expression level of TFRC in the HSC4/TFRC cells was clearly increased more than that of the parental or mock vector-transfected HSC4 cells by flow cytometry and immunoblot analysis (Fig.3B and C). Moreover, the expression level of TFRC in the SAS/shTFRC cells was significantly reduced compared with that of the parental or SAS/mock cells by flow cytometry and immunoblot analysis (Fig.3E and F), along with a reduction in the growth rate (Fig.3D). Given the combined findings of the TFRC expression and cell growth studies, TFRC is a potential cell surface marker for dysplasia and OSCC, and OSCC cell growth appears to be partly dependent on the TFRC expression rate.

### The anti-human TFRC antibody exerted direct cytotoxic effects on OSCC cells

**Figure 4:**
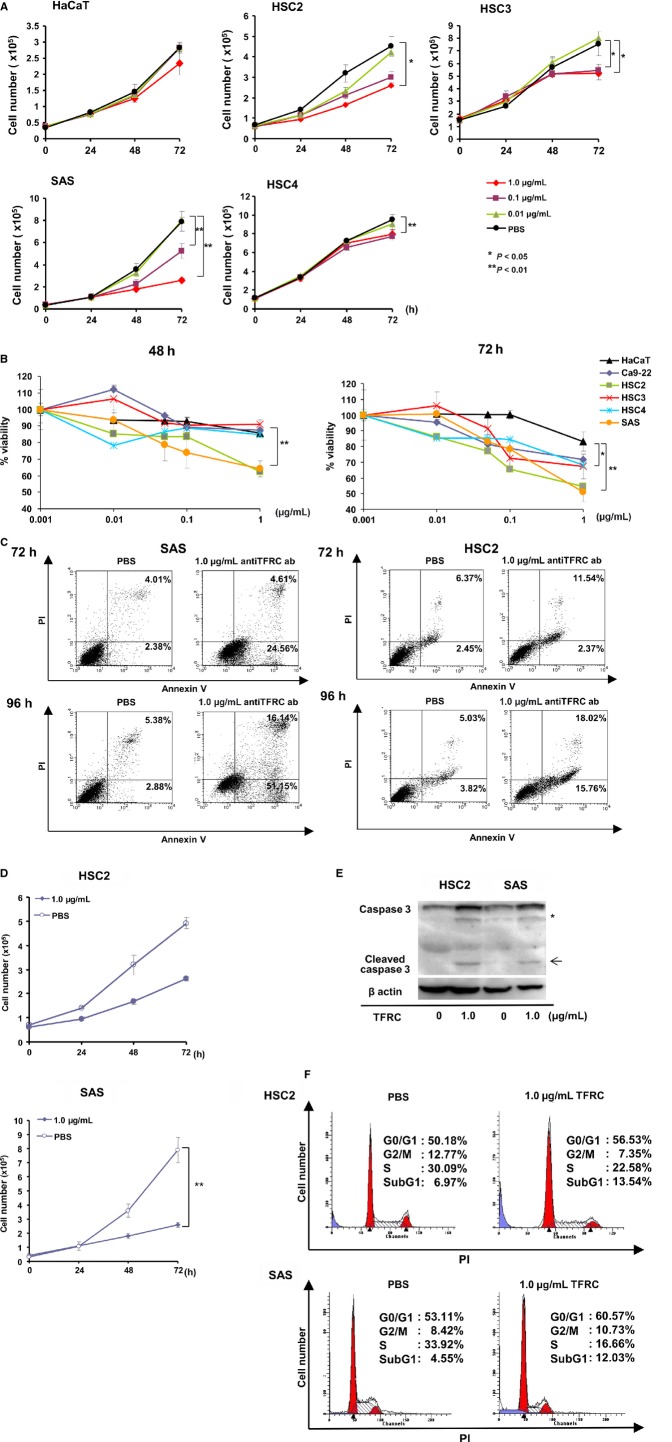
Effects of anti-TFRC antibody treatment on OSCC cells. (A) Cell growth curves of various OSCC cell lines (HSC2, HSC3, HSC4, and SAS) and control HaCaT cells after treatment with the indicated concentration of anti-TFRC antibody. The various cell lines were analyzed by MTT assay. *P < 0.05, **P < 0.01. (B) The percentages of viable cells at 48 or 72 h after treatment with the indicated concentrations of the anti-TFRC antibody were compared between the OSCC and control HaCaT cells. The experiments were performed in triplicate and repeated independently at least three times. *P < 0.05, **P < 0.01 versus HaCaT cells. (C) Following anti-TFRC antibody treatment for 72 or 96 h, SAS/OSCC and HSC2/OSCC cells were labeled with Annexin-V and propidium iodide, and the percentage of apoptotic cells was determined by flow cytometry. The experiments were performed in triplicate and repeated independently at least three times. (D) The number of viable SAS and HSC2 cells was determined at the indicated time points after treatment with 1.0 mg/mL of the anti-TFRC antibody or PBS. *P < 0.05, **P < 0.01. (E) Identification of cleaved caspase-3 (arrowheads) in OSCC cells after anti-TFRC antibody treatment. SAS/OSCC cells were treated with the anti-TFRC antibody (1.0 mg/mL) for 48 h, and immunoblotting analysis was performed with an anti-caspase-3 antibody. The asterisk indicates a nonspecific band. (F) The cell cycle phase distribution was determined for SAS and HSC2/OSCC cells at 48 h after anti-TFRC antibody treatment. The cells were labeled with propidium iodide and analyzed on a FACScan flow cytometer. OSCC, oral squamous cell carcinoma; TFRC, transferrin receptor C; MTT, methyl thiazolyl tetrazolium; PBS, phosphate-buffered saline.

Recently, we developed a method called “isolation of antigen–antibody complexes through organic solvent” (ICOS) for the comprehensive isolation of mAbs bound to cell surface molecules 16 and isolated several tumor-specific antibodies after screening many cancer cell lines by ICOS 12. Because the anti-human TFRC antibody was included among the tumor-specific antigens 13, we determined the function of the anti-human TFRC antibody against several OSCC cell lines. We initially used Cell Counting Kit 8 to determine the growth inhibitory and direct cytotoxic effects of the anti-human TFRC antibody at various concentrations and at different time points against several OSCC cell lines (Fig.4A). The cell growth rates of four OSCC cell lines (HSC2, HSC3, SAS, and HSC4) were significantly inhibited in a dose-dependent manner at 72 h after treatment, although the control HaCaT cell line was not inhibited by the anti-TFRC antibody. Concurrently, we determined the cell viability rates of the five cell lines after treatment with various concentrations of anti-TFRC antibody at each time point. The cell viabilities of all four OSCC cell lines were significantly reduced at 72 h after treatment compared to that of the HaCaT cells (Fig.4B).

To evaluate whether the increased cell death induced by the anti-TFRC antibody treatment was apoptotic cell death, we evaluated the cleaved caspase-3 band intensities and Annexin V staining of SAS and HSC2/OSCC cells after treatment with 1.0 *μ*g/mL of the anti-TFRC antibody at each time point by flow cytometry analysis. After the anti-TFRC antibody treatment, the viable SAS and HSC2 cell numbers were significantly reduced, with increased cleaved caspase-3 band intensities and higher percentages of Annexin V-positive cells (Fig.4C–E). Additionally, the anti-TFRC antibody-treated SAS and HSC2 cells exhibited higher percentages of cells in G0/G1 phase and lower percentages of cells in SubG1 phase and S phase than did the control cells (Fig.4F). Taken together, these data indicate that OSCC cell growth is suppressed by anti-TFRC antibody treatment and that apoptosis is a mechanism of this growth suppression.

### The anti-TFRC antibody-mediated ADCC against OSCC cells

**Figure 5:**
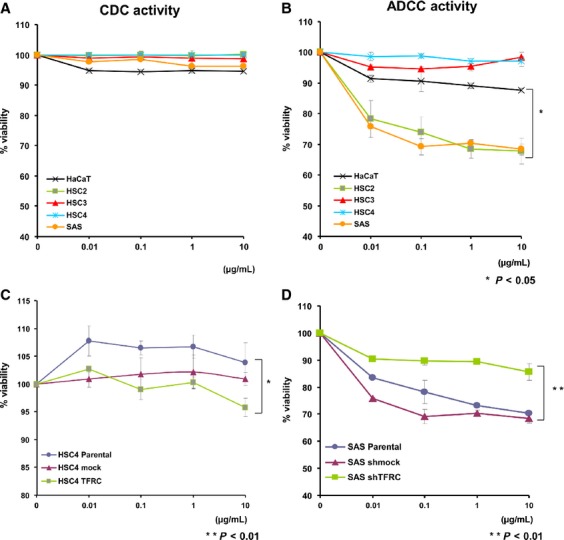
The induction of CDC and ADCC activities by anti-TFRC antibody treatment. (A) Anti-TFRC antibody-mediated CDC activity in various OSCC cells. The panel shows the percentage of viable cells. After the cells were incubated with human serum complement and anti-TFRC antibody at concentrations ranging from 0.01 to 10 mg/mL, the extent of cell lysis was measured by LDH release and is shown as the percentage of the value obtained from the untreated cells. The experiments were repeated independently at least three times. (B) Anti-TFRC antibody-mediated ADCC activity in various OSCC cells. ADCC assays were performed with the same series of cells used in (A). After incubating the cells with peripheral blood mononuclear cells from a normal donor and 0.01 to 10 mg/mL of the anti-TFRC antibody, the ADCC activity was measured by LDH release. Two OSCC cell lines (HSC2 and SAS) exhibited dose-dependent effects, whereas two other OSCC lines (HSC3 and HSC4) and control HaCaT cells showed no effects. (C) Anti-TFRC antibody-mediated ADCC activity in HSC4/TFRC cells compared with mock or parental HSC4 cells. (D) Anti-TFRC antibody-mediated ADCC activity in SAS/shTFRC cells compared with sh mock or parental SAS cells. The results are shown as the mean ± the for each sample, and all were independently repeated in triplicate. *P < 0.05, **P < 0.01. CDC, complement-dependent cytotoxicity; ADCC, antibody-dependent cellular cytotoxicity; OSCC, oral squamous cell carcinoma; TFRC, transferrin receptor C; LDH, lactate dehydrogenase.

Because the anti-TFRC antibody exerts its antitumor effects through immunological mechanisms, such as CDC and/or ADCC via its IgG Fc region, we initially investigated the CDC activity of the anti-TFRC antibody against OSCC cell lines (HSC2, HSC3, HSC4, and SAS) and the control line HaCaT. Anti-TFRC antibody treatment induced slight increases in cell death in the HaCaT and SAS cell lines, but these differences were not statistically significant (Fig.5A). Next, when evaluating the ADCC activity of the anti-TFRC antibody, the percentages of cell death of two OSCC cell lines (HSC2 and SAS) significantly increased in a dose-dependent manner, whereas two other OSCC lines (HSC3 and HSC4) and HaCaT cells showed very low percentages or no cell death (Fig.5B). Because high responder cells (HSC2 and SAS) had a relatively high expression of TFRC and low responder cells (HSC3, HSC4, and HaCaT) showed a relatively low expression, we speculated that the ADCC activity is dependent on the expression of TFRC. Therefore, we evaluated ADCC activity in shTFRC-transfected SAS cells (SAS/shTFRC) and TFRC expression vector-transfected HSC4 cells (HSC4/TFRC) compared to mock or parental cell lines. The percentages of cell death of the HSC4/TFRC cells clearly increased more than that of the mock vector-transfected or parental cells (Fig.5C), and conversely the percentages of cell death in the SAS/shTFRC cells significantly reduced compared with that of the parental or SAS/mock cells (Fig.5D).

### The anti-TFRC antibody reduced the incorporation of transferrin into cells to mediate OSCC cell death

**Figure 6:**
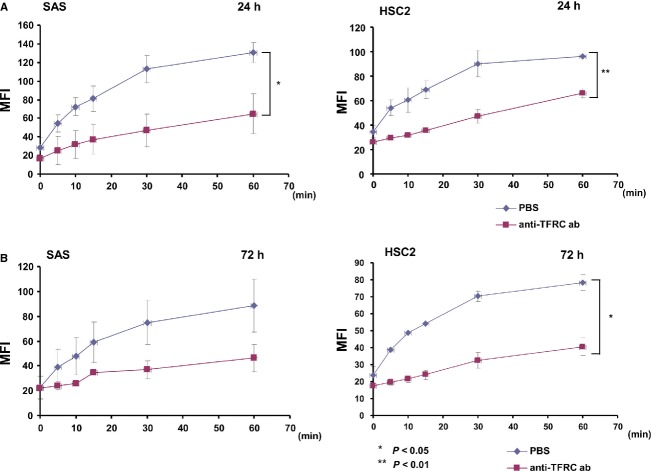
Anti-TFRC antibody treatment blocks the incorporation of transferrin into OSCC cells. Transferrin uptake in the high-TFRC-expressing SAS and HSC2 cell lines at (A) 24 h and (B) 72 h after anti-TFRC antibody treatment. Transferrin was rapidly internalized after 5 min and continuously internalized up to 60 min in the control cells, but the anti-TFRC antibody-treated cells showed inhibited transferrin incorporation. These experiments were performed with a FACScan flow cytometer, and the quantity of internalized transferrin is represented by the MFI. The left panels show the results for the SAS cells and the right panels show the results for the HSC2 cells. The experiments were performed in triplicate and repeated independently at least three times. *P < 0.05, **P < 0.01. OSCC, oral squamous cell carcinoma; TFRC, transferrin receptor C; MFI, mean fluorescence intensity.

Transferrin-bound iron is taken up via TFRC-mediated endocytosis. To evaluate the function of the anti-TFRC antibody with respect to TFRC, we next examined whether TFRC-antibody treatment inhibits the incorporation of transferrin into cells via TFRC-mediated endocytosis. After incubating SAS/OSCC cells with Alexa488-labeled transferrin, with or without the anti-TFRC antibody, the nonsurface-bound transferrin was washed away and the incorporated Alexa488-labeled transferrin was examined by flow cytometry analysis. In the OSCC cell lines SAS and HSC2, which express high levels of TFRC, transferrin was rapidly internalized after 5 min and continuously internalized until 60 min, whereas 24 h of anti-TFRC antibody treatment inhibited transferrin incorporation (Fig.6A); similar results were obtained in the SAS and HSC2 cells after 72 h of treatment (Fig.6B). Therefore, the anti-TFRC antibody reduces OSCC cell growth by inhibiting the incorporation of transferrin into cells.

### The anti-TFRC antibody exhibited antitumor effects against OSCC cells in a xenograft model in vivo

**Figure 7:**
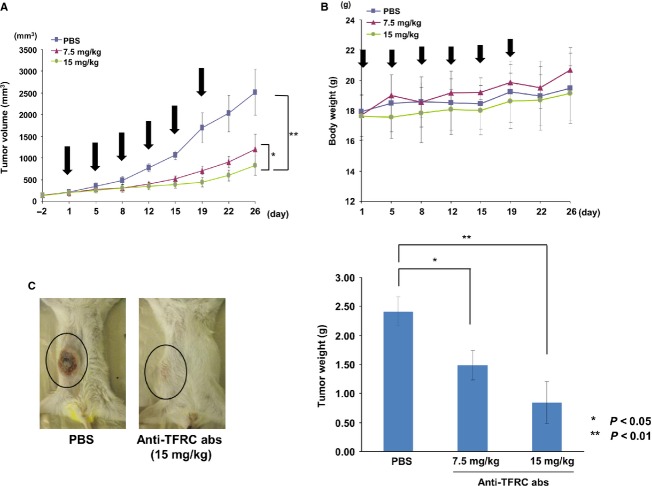
In vivo antitumor activity of the anti-TFRC antibody against OSCC in subcutaneously xenografted mice. (A) The anti-TFRC antibody reduced tumor growth in Rag-2/Jak3 double-deficient (*Rag-2*^−/−^*Jak3*^−/−^) immunodeficient mice that were xenografted with SAS cells. The anti-TFRC antibody was intravenously administered at 15 or 7.5 mg/kg twice per week for 3 weeks (arrowheads). Tumor growth was assessed by measuring the volume of each tumor at twice per week. Each group contained five mice. *P < 0.05, **P < 0.01. (B) Body weights were measured at each time point. The body weights of the treated mice gradually increased during the treatment. (C) The xenografted mice were sacrificed 29 days after treatment, and the weights of the isolated tumors from each group were directly measured. Two photos show typical cases of mice treated with PBS (left) or 15 mg/kg of the anti-TFRC antibody (right). The right panel shows the comparison of the average tumor weights ± standard errors among the three groups. *P < 0.05, **P < 0.01. OSCC, oral squamous cell carcinoma; TFRC, transferrin receptor C; PBS, phosphate-buffered saline.

To investigate the pharmacokinetics of the anti-TFRC antibody, we first intravenously injected 15 mg/kg of the anti-TFRC antibody into immunodeficient Rag-2/Jak3 double-deficient (*Rag-2*^*−/−*^*Jak3*^*−/−*^) mice 15 and measured the blood concentration of anti-TFRC antibody per day by ELISA (Fig. S2). We observed the highest blood antibody concentration immediately after administration and a reduction in about 50% (100 *μ*g/mL) of the initial concentration after 24 h. Thereafter, the blood concentration decreased slowly by about 50 *μ*g/mL until 96 h after administration. Next, to investigate the effects of the anti-TFRC antibody on OSCC cells in vivo, SAS cells were subcutaneously inoculated into the Rag-2^−/−^Jak3^−/−^ mice 15. After the tumors reached a size of 150 mm^3^, the anti-TFRC antibody or PBS (control) was intravenously injected twice per week for 3 weeks, and the tumor growth and weight were monitored at each time point (Fig.7A and B). The anti-TFRC antibody treatment significantly inhibited the growth of the SAS cell tumors in a dose-dependent manner when compared with the control (*P* < 0.05, *P* < 0.01). Additionally, the mice were sacrificed at 29 days after cell inoculation, and the tumors were weighed. The tumor weights were significantly reduced in the anti-TFRC antibody-treated mice compared to the control-treated mice (Fig.7C). And there was no obvious presentation of symptoms, suggesting that this treatment did not result in any apparent side effects. Therefore, the anti-human TFRC antibody developed by phage display is a promising candidate therapeutic antibody for OSCC patients.

## Discussion

Although up to 20% of oral dysplasia cases are thought to undergo malignant transformation to OSCC 3,4, there are no effective methods that can predict which patients with oral dysplasia will develop OSCC. Recently, several genes were identified as biomarkers of oral dysplasia and OSCC, and these genes could be useful for monitoring the development of OSCC from oral dysplasia 17–20. However, although the early diagnosis and treatment of OSCC is crucial to a more favorable prognosis, these molecules are not suitable therapeutic targets for the treatment of dysplasia and OSCC. Here, we showed that the TFRC expression level is a candidate tumor marker for OSCC development from dysplasia and that TFRC itself could possibly be a therapeutic target for OSCC with the development of the anti-human TFRC-specific antibody described in this manuscript. The mechanism behind the selective cancer cell killing ability of the anti-TFRC antibody has not yet been elucidated. However, it is speculated that cancer cells require more iron than normal cells to sustain their abnormally rapid growth rate, and reduction in the intracellular iron concentration by preventing TFRC-mediated cellular iron uptake may induce growth inhibition and cell death in cancer cells. Therefore, in future experiments, we should carefully determine the relationship between the TFRC expression time course and disease progression in cases of OSCC that have developed from dysplasia as well as the effectiveness of the anti-TFRC antibody against OSCC.

In OSCC, the anti-TFRC antibody is suspected to block the internalization of transferrin into cancer cells, and the resulting transferrin deficiency might block the cell cycle and/or induce apoptotic cell death due to the potentially abnormal transferrin requirements of cancer cells 21. Moreover, the anti-TFRC antibody might not have a significant effect on all of the OSCC cell lines because the transferrin receptor in the cells might be not completely blocked by this antibody and other pathways that are not blocked by this antibody might play a role in transferrin incorporation. The OSCC cells did not display the same kinetics of iron uptake as normal erythroblasts 22 (Fig.6). We speculate that OSCC cells require more iron than normal cells, which may be associated with higher rate of iron absorption and lower rate of iron release.

However, we showed that the anti-TFRC antibody was more effective against cancer cells that expressed high levels of TFRC than against those with low TFRC expression. High TFRC expression might indicate an increased demand for iron in OSCC cells; nevertheless, in the future, we should determine how the true iron requirement mechanism is necessary for OSCC cell growth.

Because significant progress has recently been made with regard to molecular-targeted therapy, an anti-EGFR antibody was adapted for specific OSCC therapy, and the results of this therapy indicated some good treatment outcomes 23. However, only a few antibodies or small molecules have been adapted for OSCC treatment compared to the many candidates for the treatment of stomach and colon cancer. Therefore, we are aiming to determine the usefulness of TFRC as a therapeutic target for OSCC. In particular, we isolated a series of complete human IgGs against human TFRC; these were generated using our novel strategy for isolating naïve antibodies against antigens with natural conformational epitopes on cell membranes via the phage-display method. We are now isolating several types of TFRC antibodies with which to develop molecular targeting therapies for human patients. We plan to initiate a clinical trial to examine the benefit of TFRC therapy against leukemia and other cancer types, including OSCC.

## Supporting Information

**Figure S1.** Expression of TFRC and other iron-related genes in the oral dysplasia and OSCC samples, according to microarray and RT-PCR analysis. **Figure S2.** Pharmacokinetics of the in vivo anti-TFRC antibody study and blood concentration of anti-TFRC antibody was measured per day.

**Table S1.** List of the genes within the chromosome 3q amplicon that were found to be highly expressed in the oral dysplasia and OSCC samples.
