# Supplementary material for: Development of a complete human anti-human transferrin receptor C antibody as a novel marker of oral dysplasia and oral cancer
Source: Cancer Med. 2014 Jun 2;3(4):1085–99. doi: 10.1002/cam4.267 (PMC4303177; doi:10.1002/cam4.267)
Supplement: Supplementary file 1 [file cam40003-1085-sd1.ppt]

## Slide 1
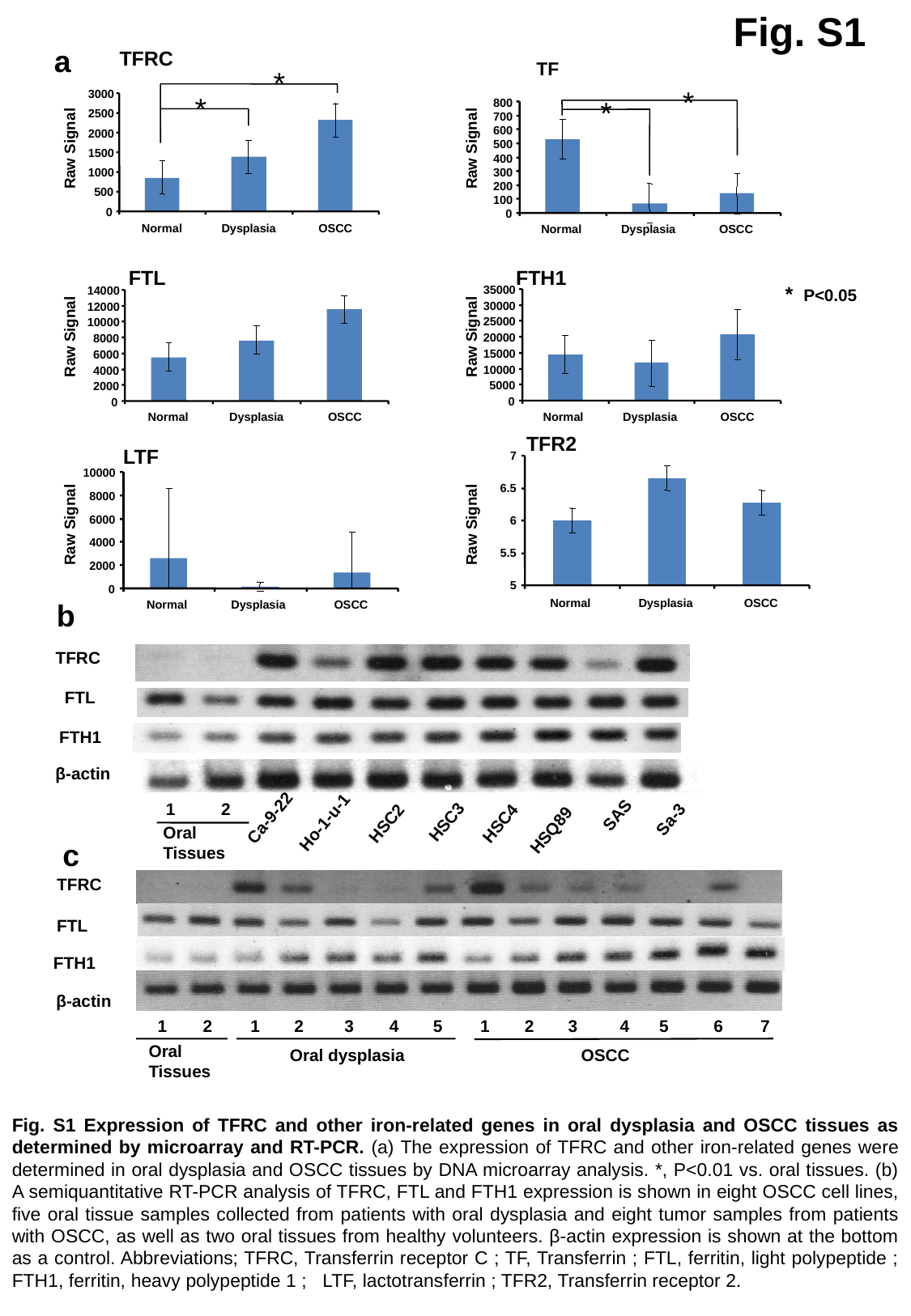

Fig. S1
a
TFRC
*
*
3000
2500
2000
1500
1000
500
0
Normal
Dysplasia
OSCC
TF
*
*
800
700
600
500
400
300
200
100
0
Normal
Dysplasia
OSCC
FTL
14000
12000
10000
8000
6000
4000
2000
0
Normal
Dysplasia
OSCC
FTH1
35000
30000
25000
20000
15000
10000
5000
0
Normal
Dysplasia
OSCC
TFR2
7
6.5
6
5.5
5
Normal
Dysplasia
OSCC
LTF
10000
8000
6000
4000
2000
0
Normal
Dysplasia
OSCC
Raw Signal
Raw Signal
Raw Signal
Raw Signal
Raw Signal
Raw Signal
* P<0.05
b
TFRC
FTL
FTH1
β-actin
1
2
Ca-9-22
SAS
Sa-3
Ho-1-u-1
HSC3
HSC4
HSC2
HSQ89
Oral
Tissues
c
TFRC
FTL
FTH1
β-actin
1
2
1
2
3
4
5
1
2
3
4
5
6
7
Oral
Tissues
Oral dysplasia
OSCC
Fig. S1 Expression of TFRC and other iron-related genes in oral dysplasia and OSCC tissues as determined by microarray and RT-PCR. (a) The expression of TFRC and other iron-related genes were determined in oral dysplasia and OSCC tissues by DNA microarray analysis. *, P<0.01 vs. oral tissues. (b) A semiquantitative RT-PCR analysis of TFRC, FTL and FTH1 expression is shown in eight OSCC cell lines, five oral tissue samples collected from patients with oral dysplasia and eight tumor samples from patients with OSCC, as well as two oral tissues from healthy volunteers. β-actin expression is shown at the bottom as a control. Abbreviations; TFRC, Transferrin receptor C ; TF, Transferrin ; FTL, ferritin, light polypeptide ; FTH1, ferritin, heavy polypeptide 1 ; LTF, lactotransferrin ; TFR2, Transferrin receptor 2.

## Slide 2
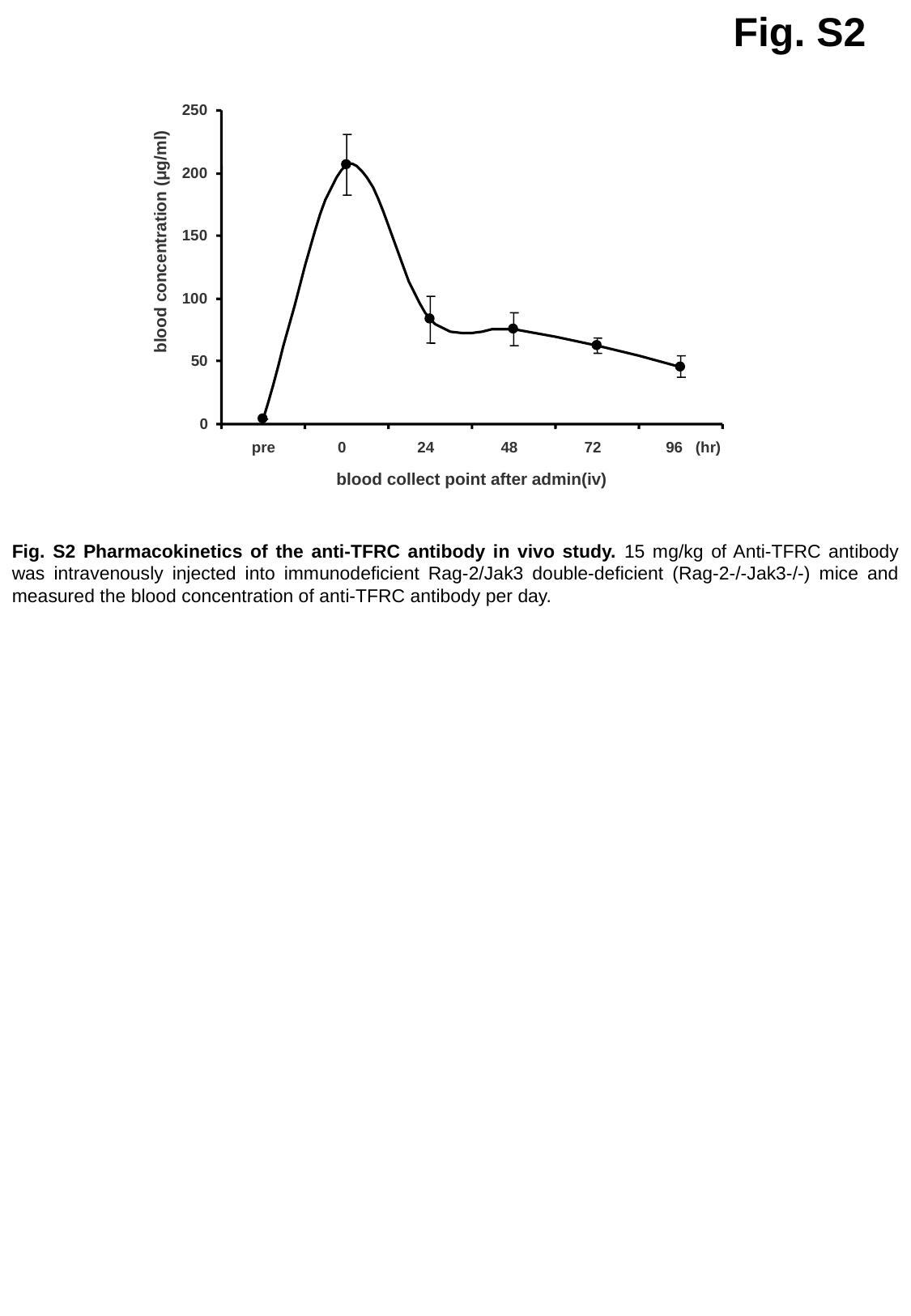

Fig. S2
250
200
150
blood concentration (μg/ml)
100
50
0
96 (hr)
pre
0
24
48
72
blood collect point after admin(iv)
Fig. S2 Pharmacokinetics of the anti-TFRC antibody in vivo study. 15 mg/kg of Anti-TFRC antibody was intravenously injected into immunodeficient Rag-2/Jak3 double-deficient (Rag-2-/-Jak3-/-) mice and measured the blood concentration of anti-TFRC antibody per day.
