# Supplementary material for: Development of a complete human anti-human transferrin receptor C antibody as a novel marker of oral dysplasia and oral cancer
Source: Cancer Med. 2014 Jun 2;3(4):1085–99. doi: 10.1002/cam4.267 (PMC4303177; doi:10.1002/cam4.267)
Supplement: Supplementary file 2 [file cam40003-1085-sd2.docx]

Table S1 List of the genes within the chromosome 3q amplicon that were found to be highly expressed in oral dysplasia and OSCC samples.

| Location | *Gene symbol* | Oral dysplasia | | OSCC | | Description | Cellular location |
| --- | --- | --- | --- | --- | --- | --- | --- |
|  |  | Fold Change | P value | Fold Change | P value |  |  |
| 3q13.2 | *PHLDB2* | 3.02 | 8.2E-05 | 5.39 | 1.5E-33 | pleckstrin homology-like domain, family B, member 2 | C, M |
| 3q13.33 | *FSTL1* | 2.52 | 2.4E-03 | 2.14 | 6.0E-14 | follistatin-like 1 | S |
| 3q21 | *MCM2* | 2.57 | 3.6E-02 | 3.25 | 3.7E-16 | minichromosome maintenance complex component 2 | N |
| 3q21.1 | *DTX3L* | 1.56 | 5.9E-03 | 2.13 | 1.9E-19 | deltex 3-like (Drosophila) | C, N |
| 3q21.1 | *PARP14* | 2.17 | 1.4E-02 | 2.98 | 2.1E-17 | poly (ADP-ribose) polymerase family, member 14 | N C |
| 3q21.1 | *PDIA5* | 1.58 | 1.3E-02 | 2.29 | 9.0E-23 | protein disulfide isomerase family A, member 5 | ER |
| 3q21.3 | *PLXNA1* | 2.01 | 3.4E-05 | 3.80 | 8.2E-29 | plexin A1 | M |
| 3q21.3 | *RUVBL1* | 1.87 | 4.3E-04 | 2.36 | 2.3E-26 | RuvB-like 1 (E. coli) | N, C, M |
| 3q22.1 | *ATP2C1* | 2.85 | 8.5E-05 | 3.46 | 4.6E-45 | ATPase, Ca++ transporting, type 2C, member 1 | G |
| 3q23 | *ATP1B3* | 2.25 | 1.6E-06 | 2.21 | 2.6E-25 | ATPase, Na+/K+ transporting, beta 3 polypeptide | M |
| 3q23 | *RBP1* | 2.61 | 8.1E-03 | 6.44 | 3.9E-23 | retinol binding protein 1, cellular | C |
| 3q24 | *GMPS* | 1.61 | 2.3E-03 | 2.17 | 3.6E-25 | guanine monphosphate synthetase | C |
| 3q24 | *CHST2* | 4.57 | 9.4E-04 | 6.29 | 4.0E-22 | carbohydrate (N-acetylglucosamine-6-O) sulfotransferase 2 | G |
| 3q24 | *CPA3* | 3.73 | 2.8E-03 | 2.89 | 1.1E-16 | carboxypeptidase A3 (mast cell) | C |
| 3q25.1 | *PFN2* | 2.21 | 5.9E-03 | 4.34 | 2.2E-19 | profilin 2 | C |
| 3q25.32 | *MLF1* | 2.70 | 4.4E-03 | 4.03 | 3.4E-19 | myeloid leukemia factor 1 | C N |
| 3q25.32 | *RSRC1* | 1.72 | 4.4E-03 | 3.14 | 5.7E-40 | arginine/serine-rich coiled-coil 1 | C, N |
| 3q26.2 | *GOLIM4* | 1.61 | 8.6E-03 | 2.19 | 1.7E-10 | golgi integral M protein 4 | G |
| 3q26.31 | *ECT2* | 1.75 | 2.4E-02 | 3.22 | 7.1E-28 | epithelial cell transforming sequence 2 oncogene | C, N |
| 3q26.31 | *FNDC3B* | 3.20 | 1.6E-03 | 6.53 | 2.7E-50 | fibronectin type III domain containing 3B | M (out) |
| 3q26.33 | *GNB4* | 1.54 | 3.4E-03 | 2.29 | 6.6E-19 | guanine nucleotide binding protein (G protein), beta polypeptide 4 | - |
| 3q27.1 | *LAMP3* | 2.30 | 6.4E-03 | 4.02 | 5.0E-16 | lysosomal-associated M protein 3 | L**,** M |
| 3q27.1 | *YEATS2* | 1.62 | 4.5E-06 | 2.06 | 3.0E-27 | YEATS domain containing 2 | N |
| 3q27.1 | *ABCC5* | 2.33 | 8.2E-03 | 2.07 | 5.7E-11 | ATP-binding cassette, sub-family C (CFTR/MRP), member 5 | M |
| 3q27.2 | *IGF2BP2* | 1.90 | 1.6E-03 | 4.66 | 9.2E-30 | insulin-like growth factor 2 mRNA binding protein 2 | C |
| 3q27.3 | *RFC4* | 1.88 | 2.4E-02 | 2.90 | 2.2E-23 | replication factor C (activator 1) 4, 37kDa | N |
| 3q27.3 | *RTP4* | 2.21 | 7.6E-03 | 4.46 | 9.0E-15 | receptor (chemosensory) transporter protein 4 | M (out) |
| 3q28 | *LEPREL1* | 3.11 | 1.6E-02 | 12.00 | 9.7E-19 | leprecan-like 1 | G |
| 3q29 | *TFRC* | 1.62 | 3.2E-02 | 2.70 | 1.6E-19 | transferrin receptor (p90, CD71) | M (out) |
| 3q29 | *RNF168* | 2.22 | 5.9E-04 | 2.19 | 1.8E-22 | ring finger protein 168, E3 ubiquitin protein ligase | N |
| 3q29 | *LRRC15* | 25.87 | 4.1E-04 | 26.29 | 1.2E-17 | leucine rich repeat containing 15 | M |
| 3q29 | *FBXO45* | 4.29 | 7.2E-05 | 3.17 | 6.1E-25 | F-box protein 45 | M, C |
| 3q29 | *FAM43A* | 4.97 | 1.6E-04 | 2.38 | 6.0E-09 | family with sequence similarity 43, member A | - |
| 3q29 | *PIGX* | 1.62 | 6.8E-03 | 2.97 | 4.3E-13 | phosphatidylinositol glycan anchor biosynthesis, class X | M, ER |
| 3q29 | *MB21D2* | 2.50 | 1.5E-03 | 3.58 | 1.0E-36 | Mab-21 domain containing 2 | - |

The indicated genes within the amplicon at chromosome 3q were picked up with more than two-fold higher expression in OSCC and 1.5-fold higher expression in oral dysplasia (p < 0.05).

M; membrane, N; nucleus, C; cytoplasm, ER; endoplasmic reticulum. M (out); outside the membrane, S; Secreted.
